# Climate change and mental health burden among caregivers in California

**DOI:** 10.1093/geront/gnag048

**Published:** 2026-04-21

**Authors:** Elizabeth Choa, David Vlahov, Sayantani Sarkar, Hermine Poghosyan

**Affiliations:** National Clinician Scholars Program, Division of General Internal Medicine and Health Services Research, David Geffen School of Medicine, University of California, Los Angeles, California, United States; Yale School of Nursing, Yale University, New Haven, Connecticut, United States; Yale School of Nursing, Yale University, New Haven, Connecticut, United States; Yale School of Public Health, Yale University, New Haven, Connecticut, United States; Yale School of Nursing, Yale University, New Haven, Connecticut, United States; Yale School of Nursing, Yale University, New Haven, Connecticut, United States

**Keywords:** Climate resilience, Caregiving burden, Health disparities

## Abstract

**Background and Objectives:**

Climate change poses a growing threat to mental health, particularly for populations already facing chronic stress. Caregivers manage multiple responsibilities under challenging conditions, yet their vulnerability to climate-related mental health impacts remains underexplored. This study assesses whether caregivers report greater climate-related mental health burden and whether structural factors are associated with this burden.

**Research Design and Methods:**

We conducted a cross-sectional analysis using 2023 California Health Interview Survey data. Multivariable logistic regressions assessed associations between caregiver status, structural factors, and climate-related mental health burden. Subgroup analysis among caregivers evaluated whether these associations were more pronounced in this population.

**Results:**

Caregivers had increased odds of climate-related mental health burden (adjusted odds ratio [aOR] = 1.57, 95% CI 1.36–1.80). Within the caregiver subgroup, higher odds were associated with housing insecurity (aOR = 1.43, 95% CI 1.11–1.84), caregiving-related financial stress (aOR = 1.54, 95% CI 1.19–2.01), bisexual/pansexual identity, (aOR = 2.96, 95% CI: 2.02–4.33), and providing care to older adults (aOR = 1.31, 95% CI 1.03–1.65). Rural caregivers in neighborhoods with low community cohesion also had elevated odds (aOR = 2.65, 95% CI 1.46–4.81).

**Discussion and Implications:**

Caregivers reported greater climate-related mental health burden than non-caregivers. Findings highlight the role of structural factors in shaping mental health and the need for climate resilience strategies that meaningfully incorporate caregiving considerations into adaptation planning. Attending to these structural determinants is essential for building resilient responses to climate change that recognize and meet the shared needs of caregivers and their care recipients.

## Background and objectives

Climate change presents a growing threat to population health worldwide (Romanello et al., 2021). Although the physical health effects of climate change are well documented, its mental health consequences remain comparatively underexplored (White et al., 2023). As climate events grow more frequent and disruptive, their impact on mental health is expected to fall disproportionately on populations already managing chronic stressors (Lawrance et al., 2022). Informal caregivers, who provide unpaid care to kin or others, may be particularly vulnerable to adverse mental health impacts from climate change (Broese van Groenou et al., 2016). Caregiving is a well-documented source of psychological strain and has been linked to increased risk for depression, anxiety, and disengagement from preventive health behaviors (Lindt et al., 2020; Nielsen et al., 2024; Sheehan et al., 2021). Although caregiving is demanding in many contexts, supporting older adults with progressive health conditions such as cognitive decline, impaired mobility, or multiple chronic illnesses can introduce additional challenges that make caregiving even more difficult (Amer Nordin et al., 2019; Riffin et al., 2017; Sheehan et al., 2021). As the population ages, both the number of caregivers and the intensity of caregiving demands are projected to rise, placing considerable pressure on individuals and the systems intended to support health (Broese van Groenou et al., 2016).

Climate change threatens to further exacerbate these demands (Romanello et al., 2021). Climate events can destabilize care routines, reduce access to essential services, and erode the community networks caregivers depend on for support (Adger et al., 2022; Romanello et al., 2021). For individuals already balancing intensive responsibilities, such disruptions can place substantial strain on mental health. Despite their central role in supporting the health and well-being of older adults, caregivers remain largely overlooked in climate–health research and are seldom integrated into climate adaptation or policy planning.

Recent climate disasters illustrate the urgency of this omission (Bouldin et al., 2018; Pickering et al., 2021). The 2018 Camp Fire in California, the state’s deadliest wildfire at the time, disproportionately impacted older adults: of the 85 fatalities, only 16 were under the age of 65 (Maranghides et al., 2023). These outcomes revealed structural failures in evacuation infrastructure and emergency communication that extend well beyond individual preparedness (Maranghides et al., 2023; White et al., 2023). In such situations, caregivers are responsible for making rapid decisions for both themselves and their care recipients, frequently under conditions of limited information, time pressure, and constrained options (Peterson et al., 2024; Pickering et al., 2021). Even so, most climate change preparedness guidance continues to emphasize individual responsibility, often with limited regard for the structural barriers that caregivers routinely face (Peterson et al., 2024).

This study was guided by an integration of structural competency, environmental health disparities, and minority stress theory, which together provide a lens to examine how climate-related stressors shape mental health outcomes. Structural competency shifts the focus from individual behaviors to the broader institutional and systemic conditions that influence mental health (Metzl & Hansen, 2014). Environmental health disparities theory positions climate change as a structural driver of health inequities, with its mental health impacts disproportionately impacting those already contending with social and economic disadvantage (Gee & Payne-Sturges, 2004). Minority stress theory further emphasizes how ongoing exposure to stressors, including discrimination and marginalization, corresponds to psychological stress (Meyer, 2003). Guided by these frameworks, we hypothesized that adults with caregiving responsibilities, especially those experiencing financial stress, housing insecurity, or rural isolation, would be more likely to report climate-related mental health burden. We further anticipated that this burden would be more commonly reported among LGBQ+ individuals and among those living in contexts of structural disadvantage, such as neighborhoods with low community cohesion.

Although caregivers play a critical role during climate disruptions, data are sparse on how caregiving itself may be associated with vulnerability to climate-related mental health impacts. This study addresses this gap through two objectives. First, we assessed whether caregiver status is associated with greater climate-related mental health burden. Second, we examined whether structural factors are associated with climate-related mental health burden, and whether these associations differ for caregivers. A clearer understanding of these layered pressures is essential for informing climate resilience strategies that include caregivers as key stakeholders. Findings from this study can help advance age-inclusive adaptation planning by more effectively integrating caregiver needs into emergency preparedness and climate resilience efforts.

## Research design and methods

### Data source

We conducted a cross-sectional analysis of data from the 2023 California Health Interview Survey (CHIS), the nation’s largest state-level population health survey. We analyzed data collected between January 1 and December 31, 2023, as this survey cycle was the first to include questions on both climate change and caregiving. CHIS employs an address-based sampling design and gathers data through a combination of web and telephone interviews. The survey collects self-reported information on health behaviors and health care needs from a representative sample of non-institutionalized California residents (California Health Interview Survey, 2024a). The adult response rate was 64.7% (California Health Interview Survey, 2023). CHIS adheres to a rigorous consent process to ensure participants are fully informed and assured of the confidentiality of their responses. This study was determined to be exempt by the Yale University Institutional Review Board and followed STROBE reporting guidelines.

### Sample

In 2023, a total of 21,671 adults (aged ≥18 years) completed the CHIS. Of these, 14,319 reported experiencing a climate event and were included in this study. After excluding 12 individuals with missing data on key variables, the final analytic sample comprised 14,307 respondents. Among them, 4,417 identified as caregivers for subgroup analysis.

### Measures

#### Outcome

Climate-related mental health burden was the outcome variable that was measured with a series of multipart questions. All survey participants were asked, “In the past two years, have you or members of your household personally experienced: Extreme heat wave? Wildfire? Smoke from wildfire? Flood/rising sea levels/mudslide?” with response options of “yes” and “no.” Participants who experienced any of these events were subsequently asked, “Was your mental health (or the mental health of members of your household) harmed by any of these events?” Those who answered “yes” were categorized as having experienced climate-related mental health burden.

#### Caregiving variables

Caregiving status served as the primary independent variable. Participants were classified as caregivers if they reported providing short- or long-term care in the past 12 months to a family member or friend with a serious or chronic illness or disability. Among caregivers, we examined two caregiving-specific variables. Caregiving financial stress was assessed using the question, “How much of a financial stress would you say that caring for your [care recipient] is/was for you?” Respondents who selected “extremely stressful” or “somewhat stressful” were categorized as experiencing financial stress related to caregiving. Caregiving for an older adult was identified when participants selected “old age, aging” in response to a question about the care recipient’s illness or disability.

#### Covariates

Covariates were selected to capture two key domains that may influence climate-related mental health: (1) individual-level factors established in prior research as relevant to mental health and (2) less-studied structural or community factors that may shape climate vulnerability. The first domain included age (18–39, 40–59, 60+ years), gender (female or male), race and ethnicity (Hispanic, non-Hispanic Asian, non-Hispanic Black, non-Hispanic White, or other/≥2 races), educational attainment (high school diploma or less; some college, vocational school, or Associate’s degree; college degree or higher), marital status (never married, married/living with partner, widowed/separated/divorced), employment status (full time, part time, unemployed), household income (0%–200% or >200% federal poverty level), general health (poor/fair, good, very good/excellent), and psychological distress in the past 12 months (yes or no). Race and ethnicity were included to reflect differences in exposure to social drivers influencing mental health.

The second domain included rurality (urban vs. rural), community cohesion (based on agreement with the statement “People in my neighborhood are willing to help their neighbors”; categorized as agree/strongly agree vs. disagree/strongly disagree), and sexual orientation (lesbian or gay, bisexual or pansexual, straight, or other). Responses of “Don’t know,” “I use a different term,” or “Prefer not to answer” for sexual orientation were grouped under “other” to capture the wide range of queer+ identities, including those who are questioning, exploring, or choosing not to disclose their identity. Housing insecurity was measured by asking respondents how often they worried about paying for rent or mortgage. Those who selected “very often” or “somewhat often” were classified as experiencing housing insecurity.

### Statistical analysis

Weighted frequencies were calculated for the full analytic sample and caregiver subgroup, and *χ*^2^ tests were used to assess bivariable associations. We first used multivariable logistic regression with the full analytic sample to examine the association between caregiver status and climate-related mental health burden, adjusting for previously described covariates. An interaction term between rurality and community cohesion was included to assess for the potential synergistic effects of rurality and community cohesion on mental health. A separate subgroup analysis was then conducted among caregivers only, adding caregiver-specific predictors and retaining the prespecified interaction term. All models estimated odds ratios (ORs) with 95% CIs. Statistical significance was defined as a 2-sided *p* < .05. Analyses accounted for the complex survey design using the *svy* suite in Stata, version 19 (StataCorp LLC), with variance estimation based on the jackknife method (California Health Interview Survey, 2024b).

## Results

Table 1 displays the characteristics of participants who experienced a climate event and the caregiver subgroup within this population. After applying sample weights, the 14,307 respondents represented an estimated 17,861,613 California adults who experienced a climate event. Among this population, 50.8% were female; 41.1% were aged 18–39 years; 34.9% identified as Hispanic, and 42.0% reported housing insecurity. Among the 4,417 caregivers, 56.9% were female; 34.7% were aged 18–39 years; and 33.5% identified as Hispanic. Compared with non-caregivers, caregivers were more likely to be over age 40 (*p* < .001), female (*p* < .001), non-Hispanic Black (*p* < .001), lesbian/gay or bisexual/pansexual (*p* < .01), and living in rural areas (*p* < .05). Housing insecurity was also reported more frequently among caregivers (45.8%) than non-caregivers (40.5%, *p* < .001).

**Table 1:** Characteristics of adults, caregivers, and non-caregivers who experienced a climate event—(California Health Interview Survey, 2023).^a^

Characteristics	Full adult sample (*N* = 14,307)		Caregiver subgroup (*n* = 4,417)		Non-caregivers (*n* = 9,890)	
	Unweighted No.	Weighted % (95% CI)	Unweighted No.	Weighted % (95% CI)	Unweighted No.	Weighted % (95% CI)
**Structural, community level**						
**Caregiver**						
No	9,890	71.3 (70.2–72.3)	–	–	–	–
Yes	4,417	28.7 (28.7–29.8)	–	–	–	–
**Caregiver financial stress**						
Low	–	–	3,385	75.6 (73.5–77.5)	–	–
High	–	–	1,032	24.4 (22.5–26.5)	–	–
**Caring for an older adult**						
No	–	–	2,727	62.0 (59.7–64.3)	–	–
Yes	–	–	1,690	38.0 (35.7–40.3)	–	–
Rurality*						
Urban	11,198	87.4 (86.7–87.9)	3,427	86.3 (85.1–87.4)	7,771	87.8 (87.0–88.5)
Rural	3,109	12.6 (12.1–13.3)	990	13.7 (12.6–14.9)	2,119	12.2 (11.5–13.0)
Housing insecurity***						
Low	8,874	58.0 (56.7–59.2)	2,550	54.2 (52.1–56.3)	6,324	59.5 (58.0–60.9)
High	5,433	42.0 (40.8–43.3)	1,867	45.8 (43.7–47.9)	3,566	40.5 (39.1–42.0)
**Community cohesion**						
High	12,246	82.1 (81.1–83.0)	3,771	83.1 (81.4–84.8)	8,475	81.6 (80.6–82.7)
Low	2,061	17.9 (17.0–18.9)	646	16.9 (15.2–18.6)	1,415	18.4 (17.3–19.4)
Sexual orientation**						
Straight	12,416	83.6 (82.7–84.5)	3,818	83.1 (81.5–84.5)	8,598	83.8 (82.7–84.9)
Lesbian or gay	562	3.8 (3.5–4.2)	176	4.0 (3.4–4.8)	386	3.8 (3.3–4.2)
Bisexual or pansexual	704	6.5 (5.9–7.2)	244	8.0 (6.9–9.3)	460	5.9 (5.3–6.7)
Other	625	6.1 (5.5–6.7)	179	4.9 (4.1–6.0)	446	6.5 (5.8–7.3)
**Individual level**						
Age group***						
18–39	3,331	41.1 (40.2–42.0)	805	34.7 (32.8–36.6)	2,526	43.7 (42.5–44.9)
40–59	5,121	30.2 (29.2–31.1)	1,690	32.7 (30.9–34.7)	3,431	29.1 (27.9–30.3)
60+	5,855	28.7 (28.0–29.5)	1,922	32.6 (30.9–34.3)	3,933	27.2 (26.2–28.2)
Race/ethnicity***						
White, NH	7,793	44.0 (43.3–44.8)	2,370	43.9 (41.9–45.9)	5,423	44.1 (42.9–45.3)
Hispanic	3,590	34.9 (34.1–35.7)	1,144	33.5 (31.7–35.5)	2,446	35.4 (34.3–36.6)
African American, NH	540	4.8 (4.4–5.3)	195	6.8 (5.8–7.9)	345	4.0 (3.5–4.6)
Asian only, NH	1,708	13.4 (12.8–14.0)	455	12.2 (11.0–13.6)	1,253	13.9 (13.1–14.7)
Other/two or more races	676	2.9 (2.7–3.0)	253	3.6 (3.0–4.3)	423	2.6 (2.3–2.8)
Educational attainment***						
Highschool or less	2,087	31.5 (30.6–32.4)	554	28.0 (26.3–30.0)	1,533	32.9 (31.7–34.0)
Some college or vocational	4,025	21.2 (20.3–22.1)	1,343	23.6 (22.0–25.3)	2,682	20.2 (19.1–21.5)
College or higher	8,195	47.3 (46.4–48.3)	2,520	48.4 (46.4–50.3)	5,675	46.9 (45.7–48.2)
Marital status**						
Never married	2,936	27.9 (26.9–28.9)	852	24.3 (22.2–26.5)	2,084	29.3 (28.1–30.6)
Married/living with partner	8,140	58.4 (57.3–59.6)	2,554	60.8 (58.6–62.9)	5,586	57.5 (56.2–58.9)
Widow, separated or divorced	3,231	13.7 (13.0–14.4)	1,011	14.9 (13.6–16.3)	2,220	13.2 (12.3–14.0)
Sex assigned at birth***						
Male	6,024	49.2 (48.6–49.9)	1,612	43.1 (41.2–44.9)	4,412	51.7 (50.7–52.7)
Female	8,283	50.8 (50.1–51.4)	2,805	56.9 (55.1–58.8)	5,478	48.3 (47.3–49.3)
**Employment**						
Full time (21+ hr/week)	7,492	54.8 (53.7–56.0)	2,262	53.4 (51.3–55.5)	5,230	55.4 (53.9–56.8)
Part time (0–20 hr/week)	1,241	8.9 (8.2–9.7)	407	9.0 (7.8–10.3)	834	8.9 (8.0–9.9)
Unemployed	5,574	36.3 (35.2–37.4)	1,748	37.6 (35.7–39.6)	3,826	35.7 (34.3–37.11)
**Household income**						
Below 200% FPL	3,364	26.7 (25.4–28.0)	1,092	27.4 (25.2–29.7)	2,272	26.4 (25.0–27.9)
200% FPL and above	10,943	73.3 (71.9–74.5)	3,325	72.6 (70.3–74.8)	7,618	73.6 (72.2–75.0)
Psychological distress**						
None in last year	12,316	83.1 (82.0–84.1)	3,726	81.0 (79.4–82.5)	8590	83.9 (82.6–85.1)
Yes in last year	1,991	16.9 (15.9–18.0)	691	19.0 (17.5–20.6)	1300	16.1 (14.9–17.4)
**General health status**						
Fair–poor	2,348	16.9 (15.8–18.0)	731	17.9 (16.2–19.7)	1,617	16.5 (15.2–17.8)
Good	4,471	32.1 (30.9–33.3)	1,442	33.4 (31.4–35.4)	3,029	31.6 (30.3–33.0)
Excellent–very good	7,488	51.0 (49.7–52.3)	2,244	48.7 (46.6–50.8)	5,244	51.9 (50.3–53.4)

*Note.* NH = non-Hispanic; FPL = federal poverty level; N = analytic sample of adults who experienced a hazardous climate event; *n* = subgroup sizes (caregivers and non-caregivers).

a Unweighted frequencies and weighted percentages (95% CI). *p* values based on weighted comparisons between caregivers and non-caregivers within each category.

* *p* < .05,

** *p* < .01,

*** *p* < .001.

Overall, 23.5% of participants reported climate-related mental health burden (Table 2). In the full sample, this burden was more prevalent among younger adults (18–39 years: 28.8%), individuals experiencing housing insecurity (27.8%), and caregivers (30.0%) (all *p* < .001). Higher prevalence was also observed among participants living in rural areas, those in neighborhoods with low community cohesion, and individuals identifying as bisexual/pansexual or lesbian/gay (all *p* < .001).

**Table 2:** Prevalence of climate-related mental health harm among adults, caregivers, and non-caregivers—(California Health Interview Survey, 2023).^a^

Characteristics	Full adult sample	Caregiver subgroup	Non-caregivers
**Mental health harmed by climate events**	23.5 (22.6–24.5)	30.0 (27.8–32.3)	20.94 (19.9–22.1)
**Structural, community level**			
**Caregiver**	***		
No	20.9 (19.9–22.1)	–	–
Yes	30.0 (27.8–32.3)	–	–
**Caregiver financial stress**		***	
Low	–	26.7 (24.2–29.3)	–
High	–	40.3 (35.9–44.8)	–
**Caring for an older adult**		*	
No	–	27.7 (25.1–30.4)	–
Yes	–	33.8 (30.0–37.9)	–
** Rurality**	***	**	***
Urban	22.8 (21.8–23.8)	29.0 (26.7–31.4)	20.3 (19.2–21.4)
Rural	28.8 (26.4–31.4)	36.2 (31.4–41.3)	25.5 (22.8–28.5)
** Housing insecurity**	***	***	***
Low	20.4 (19.3–21.6)	25.1 (22.8–27.6)	18.7 (17.3–20.1)
High	27.8 (26.0–29.7)	35.8 (32.2–39.5)	24.2 (22.0–26.6)
**Community cohesion**	***		***
High	22.4 (21.4–23.4)	29.3 (26.9–31.7)	19.6 (18.5–20.7)
Low	28.9 (26.0–31.9)	33.6 (28.7–39.0)	27.1 (23.6–31.0)
**Sexual orientation**	***	***	***
Straight	21.5 (20.5–22.5)	26.8 (24.6–29.1)	19.4 (18.2–20.6)
Lesbian or gay	29.2 (24.3–34.6)	33.8 (26.9–41.5)	27.3 (21.1–34.4)
Bisexual or pansexual	49.2 (44.5–53.8)	62.5 (54.5–69.8)	41.9 (36.4–47.7)
Other	20.9 (17.4–24.9)	28.3 (20.8–37.2)	18.6 (14.9–23.0)
**Individual level**			
** Age group**	***	***	***
18–39	28.8 (27.2–30.6)	38.5 (24.8–42.5)	25.7 (23.8–27.7)
40–59	20.8 (19.1–22.6)	24.4 (21.3–27.8)	19.1 (17.1–21.3)
60+	18.9 (17.6–20.2)	26.5 (23.9–29.2)	15.2 (13.8–16.7)
** Race/ethnicity**	***	***	***
White, NH	27.2 (25.8–28.6)	34.1 (31.3–36.9)	24.4 (22.9–26.0)
Hispanic	20.2 (18.4–22.3)	27.5 (23.9–31.5)	17.5 (15.4–19.7)
African American, NH	21.2 (16.1–27.3)	22.2 (13.9–33.5)	20.5 (14.6–27.9)
Asian Only, NH	19.9 (17.6–22.5)	25.2 (21.0–29.9)	18.1 (15.4–21.1)
Other/two or more races	28.8 (23.7–34.6)	34.4 (24.1–46.4)	25.7 (20.6–31.6)
** Educational attainment**	***		***
Highschool or less	18.2 (16.2–20.4)	27.5 (23.2–32.2)	15.1 (12.7–17.7)
Some college or vocational	24.0 (22.1–26.1)	31.8 (27.5–36.4)	20.4 (18.2–22.8)
College or higher	26.9 (25.7–28.0)	30.6 (28.0–33.3)	25.3 (23.9–26.7)
** Marital status**	**	*	**
Never married	24.9 (22.8–27.1)	34.5 (29.6–39.8)	21.6 (19.4–24.0)
Married/living with partner	23.9 (22.5–25.3)	29.3 (26.4–32.3)	21.6 (20.1–23.2)
Widow, separated or divorced	19.3 (17.2–21.6)	25.6 (20.9–30.9)	16.4 (14.4–18.6)
** Sex assigned at birth**	***		**
Male	21.4 (19.8–23.2)	27.3 (23.5–31.4)	19.5 (17.8–21.2)
Female	25.6 (24.5–26.7)	32.1 (29.4–34.8)	22.5 (21.3–23.8)
** Employment**	*		***
Full time (21+ hr/week)	24.7 (23.5–26.0)	29.5 (26.6–32.6)	22.9 (21.5–24.3)
Part time (0–20 hr/week)	22.7 (20.0–25.8)	29.9 (24.0–36.6)	19.8 (16.6–23.5)
Unemployed	21.9 (20.3–23.6)	30.7 (27.5–34.1)	18.2 (16.4–20.1)
** Household income**		*	**
Below 200% FPL	22.9 (21.0–24.9)	33.7 (29.6–38.0)	18.4 (16.2–20.8)
200% FPL and above	23.8 (22.8–24.8)	28.6 (26.2–31.2)	21.9 (20.8–23.0)
** Psychological distress**	***	***	***
None in last year	19.5 (18.6–20.5)	24.6 (22.4–26.8)	17.6 (16.5–18.7)
Yes in last year	43.3 (40.3–46.3)	53.2 (47.8–58.6)	38.6 (34.8–42.5)
** General health status**	*		
Fair–poor	25.7 (23.6–28.0)	33.3 (28.2–38.7)	22.4 (19.7–25.3)
Good	23.8 (21.7–25.9)	30.1 (26.4–34.1)	20.4 (19.1–21.8)
Excellent–very good	22.7 (21.6–23.8)	28.7 (26.1–31.6)	21.1 (18.9–23.4)

*Note.* NH = non-Hispanic; FPL = federal poverty level.

a All values represent weighted prevalence with 95% confidence intervals. *p* values are based on weighted comparisons within each category.

* *p* < .05,

** *p* < .01,

*** *p* < .001.

Within the caregiver subgroup, climate-related mental health burden was highest among bisexual/pansexual individuals (62.5%), those experiencing caregiving financial stress (40.3%), and individuals aged 18–39 years (38.5%) (all *p* < .001). Compared to non-caregivers, caregivers had higher prevalence of climate-related mental health burden among those identifying as bisexual/pansexual (62.5% vs. 41.9% for non-caregivers, both *p* < .001), living in rural areas (36.2%, *p* < .01 vs. 25.5% for non-caregivers, *p* < .001), and experiencing housing insecurity (35.8% vs. 24.2% for non-caregivers, both *p* < .001).

Logistic regression results for the full sample indicated that caregiver status was independently associated with climate-related mental health burden. In the adjusted full-sample model (Table 3), higher odds of reporting climate-related mental health burden was observed among caregivers (adjusted odds ratio [aOR] = 1.57, 95% CI 1.36–1.80, *p* < .001]; individuals identifying as lesbian/gay or bisexual/pansexual (aOR = 1.37, 95% CI 1.01–1.85 and aOR = 2.26, 95% CI 1.79–2.85, respectively, *p* < .001); younger adults (aOR = 1.47, 95% CI 1.24–1.73, *p* < .001); rural residents (aOR = 1.54, 95% CI 1.30–1.82, *p* < .001); individuals experiencing housing insecurity (aOR = 1.34, 95% CI 1.17–1.54, *p* < .001); and those living in neighborhoods with low community cohesion (aOR = 1.26, 95% CI 1.04–1.52, *p* < .05). The interaction term was not statistically significant in the full sample model.

**Table 3:** Multivariable logistic regression of factors associated with climate-related mental health harm among adults who experienced climate event—(California Health Interview Survey, 2023).^a^

Adult sample	Adjusted, OR (95% CI)
**Structural, community level**	
**Caregiver**	***
No	1
Yes	1.57 (1.36–1.80)
**Community cohesion**	*
High	1
Low	1.26 (1.04–1.52)
** Rurality**	***
Urban	1
Rural	1.54 (1.30–1.82)
Community cohesion rurality*	
Low rural*	1.00 (0.67–1.47)
**Housing insecurity**	***
Low	1
High housing insecurity	1.34 (1.17–1.54)
** Sexual orientation**	***
Straight	1
Lesbian or gay	1.37 (1.01–1.85)
Bisexual or pansexual	2.26 (1.79–2.85)
Other	0.98 (0.76–1.26)
**Individual level**	
** Age group**	***
40–59	1
18–39	1.47 (1.24–1.73)
60+	0.93 (0.79–1.09)
** Race**	***
White, NH	1
Hispanic	0.58 (0.50–0.69)
African American, NH	0.71 (0.50–1.01)
Asian Only, NH	0.58 (0.48–0.70)
Other/two or more races	0.93 (0.69–1.27)
** Educational attainment**	***
Highschool or less	1
Some college or vocational	1.24 (1.05–1.46)
College or higher	1.55 (1.30–1.86)
** Marital status**	**
Never married	1
Married/living with partner	1.34 (1.12–1.62)
Widow, separated or divorced	1.10 (0.91–1.35)
** Sex assigned at birth**	*
Male	1
Female	1.14 (1.01–1.29)
** Employment status**	
Full time (21+ hr/week)	1
Part time (0–20 hr/week)	0.94 (0.77–1.14)
Unemployed	0.99 (0.85–1.15)
** Household income**	
Below 200% FPL	1
200% FPL and above	0.96 (0.82–1.12)
**Psychological distress**	***
None in last year	1
Yes in last year	2.38 (2.03–2.79)
**General health**	
Fair–poor	1
Good	0.89 (0.75–1.05)
Excellent–very good	0.83 (0.71–0.96)

*Note.* NH = non-Hispanic; FPL = federal poverty level.

a Model was adjusted for all covariates listed. aORs are presented with 95% CIs.

* *p* < .05,

** *p* < .01,

*** *p* < .001.

In the caregiver subgroup (Table 4), several predictors showed patterns similar to the adjusted full-sample model. Younger age (aOR = 1.65, 95% CI 1.29–2.10, *p* < .001); bisexual/pansexual identity (aOR = 2.96, 95% CI 2.02–4.33, *p* < .001); and housing insecurity (aOR = 1.43, 95% CI 1.11–1.84, *p* < .01) remained associated with higher odds of reporting climate-related mental health burden. Two caregiver-specific factors were also significantly associated with the outcome: caregiving-related financial stress (aOR = 1.54, 95% CI 1.19–2.01, *p* < .01); and caregiving for older adults (aOR = 1.31, 95% CI 1.03–1.65, *p* < .05). In contrast to the adjusted full-sample model, neither rural residence nor community cohesion was independently associated with climate-related mental health burden in the caregiver subgroup. Their interaction, however, was statistically significant (aOR = 2.65, 95% CI 1.46–4.81, *p* < .01), indicating elevated odds among caregivers living in rural areas with low community cohesion compared with those in urban areas.

**Table 4:** Multivariable logistic regression of factors associated with climate-related mental health harm among caregivers who experienced climate event—California Health Interview Survey (2023)^a^.

Caregiver subgroup	Adjusted, OR (95% CI)
**Structural, community level**	
**Caregiver financial stress**	**
Low	1
High	1.54 (1.19–2.01)
**Caregiver recipient old age**	*
No	1
Yes	1.31 (1.03–1.65)
**Community cohesion**	
High	1
Low	0.82 (0.61–1.11)
**Rurality**	***
Urban	1
Rural	1.37 (0.99–1.91)
Community cohesion rurality*	**
Low rural*	2.65 (1.46–4.81)
**Housing insecurity**	**
Low	1
High housing insecurity	1.43 (1.11–1.84)
**Sexual orientation**	***
Straight	1
Lesbian or gay	1.31 (0.89–1.94)
Bisexual or pansexual	2.96 (2.02–4.33)
Other	0.89 (0.53–1.51)
**Individual level**	
**Age group**	***
40–59	1
18–39	1.65 (1.29–2.10)
60+	1.28 (1.0–1.64)
** Race**	***
White, NH	1
Hispanic	0.56 (0.44–0.71)
African American, NH	0.62 (0.35–1.08)
Asian Only, NH	0.59 (0.44–0.79)
Other/two or more races	0.76 (0.42–1.38)
** Educational attainment**	
High school or less	1
Some college or vocational	1.05 (0.80–1.37)
College or higher	1.27 (0.97–1.67)
** Marital status**	
Never married	1
Married/living with partner	1.24 (0.86–1.78)
Widow, separated or divorced	0.98 (0.66–1.46)
** Sex assigned at birth**	
Male	1
Female	1.22 (0.96–1.56)
** Employment status**	
Full Time (21+ hr/week)	1
Part Time (0–20 hr/week)	1.06 (0.77–1.45)
Unemployed	0.99 (0.78–1.24)
** Household income**	
Below 200% FPL	1
200% FPL and Above	0.79 (0.60–1.05)
** Psychological distress**	***
None in last year	1
Yes in last year	2.51 (1.84–3.43)
** General health**	
Fair–poor	1
Good	0.98 (0.73–1.31)
Excellent–very good	0.95 (0.70–1.30)

*Note.* NH = non-Hispanic; FPL = federal poverty level.

a Model was adjusted for all covariates listed. aORs are presented with 95% CI.

* *p* < .05,

** *p* < .01,

*** *p* < .001.

## Discussion and implications

In this study of California adults, caregivers had higher odds of reporting climate-related mental health burden than non-caregivers, even after accounting for individual and structural characteristics. Climate-related mental health burden was also more frequently reported among adults experiencing housing insecurity, living in rural areas, and residing in neighborhoods with low community cohesion. These patterns provide important context for understanding how place-based and structural conditions may relate to climate vulnerability across populations.

### Caregivers, financial strain, and caring for older adults

Caregiver status emerged as a key correlate of climate-related mental health burden in this study. The prominence of caregiving status in these results aligns with extensive scholarship documenting the emotional, financial, and logistical demands of caregiving and the persistent lack of structural supports to meet those demands (Bevans & Sternberg, 2012; Bouldin et al., 2018; Liu et al., 2019). Despite their central role in supporting older adults, caregivers remain largely absent from climate–health discourse (Bailie et al., 2022). Whereas older adults and individuals with chronic conditions are widely recognized within climate planning (Romanello et al., 2021), caregivers themselves are less often considered, even though the conditions under which caregiving occurs can influence how vulnerable individuals experience climate disruptions.

Caregiving-specific findings further illuminate these structural contexts. Caregivers reporting financial stress or housing insecurity had higher odds of reporting climate-related mental health burden, echoing evidence that caregiving can generate long-term economic vulnerability, including forgone earnings, reduced career mobility, and cumulative financial strain (Heitmueller & Inglis, 2007; Lahaie et al., 2013). These pressures can narrow the strategies available to caregivers when climate events introduce sudden disruptions. These challenges are often intensified for caregivers working in lower-wage or precarious jobs who often face workplace constraints that limit their capacity to respond to crises, such as limited access to paid caregiver leave or concerns about employer retaliation for caregiving needs (Heitmueller & Inglis, 2007; Heymann et al., 2024; Lahaie et al., 2013). Such conditions can intensify financial strain and restrict the resources available during emergencies.

Caring for older adults also warrants particular attention. Supporting individuals living with dementia, multimorbidity, or mobility limitations typically requires highly structured routines and substantial emotional and logistical labor (Fakeye et al., 2023). Climate events that disrupt transportation, communication systems, or formal care services may make it especially difficult for caregivers in these situations to maintain continuity of care. These caregiving-specific patterns underscore the importance of considering both structural conditions and the demands of caring for older adults when assessing climate-related mental health burden among caregivers. Care contexts that involve intensive or complex needs may offer fewer buffers against additional stressors, which is consistent with the elevated levels of mental health burden reported by these caregivers.

### The limits of individual preparedness

These disparities are unlikely to reflect individual shortcomings. Instead, they parallel broader gaps in policy, infrastructure, and systems that influence how people prepare for, respond to, and recover from climate events (Himes-Cornell et al., 2018). Much of the existing literature focuses on care recipients or individual-level preparedness (Fazeli et al., 2024). Although this work has advanced understanding of personal readiness, it provides limited insight into the structural constraints related to caregiver wellbeing in the context of climate change (Pickering et al., 2021). For example, caregivers may avoid public shelters due to concern that these environments cannot accommodate individuals with dementia or complex medical needs (Peterson et al., 2024). Such decisions often reflect limitations in inclusive infrastructure rather than lack of personal readiness and illustrate how systemic design gaps can constrain caregiver options during emergencies (Peterson et al., 2024). The present findings align with this broader pattern, highlighting the need to move beyond individual-focused approaches to consider the structural conditions under which caregiving occurs.

### Rurality, community cohesion, and the amplification of climate-related mental health burden

These challenges appear particularly acute in rural and socially fragmented communities. The significant interaction between rural residence and low community cohesion suggests that these structural conditions may jointly relate to the heightened burden caregivers reported. The 2018 Camp Fire in Northern California illustrates how these dynamics can unfold. The fire damaged power lines, communication systems, and roads—conditions characteristic of rural infrastructural vulnerabilities (Maranghides et al., 2023). At the same time, it disrupted interpersonal networks that residents commonly relied on for information sharing and evacuation support (Chase & Hansen, 2021; Maranghides et al., 2023). In rural areas with limited formal services, such networks often function as an essential layer of support for caregivers. During the Camp Fire, however, the rapid spread of the fire, communication breakdowns, and blocked evacuation routes constrained the ability of these informal supports to mobilize, leaving some older adults and individuals with mobility limitations without timely assistance. In some cases, caregivers could not reach them due to emergency re-entry restrictions (Butte County District Attorney’s Office, 2020).

This convergence mirrors patterns observed in our findings. When rural conditions coincide with low community cohesion, caregivers may have fewer viable options for ensuring safety during climate disruptions. The example reinforces a broader insight: individual preparedness, while valuable, may be insufficient when structural and social systems falter (Costello et al., 2009). These patterns are consistent with a growing body of evidence on systemic disadvantage and suggest that the cumulative social and environmental inequities may contribute to the mental health burdens caregivers report during climate events (Dennin et al., 2025; White et al., 2023).

### Climate-related mental health burden among bisexual, pansexual, and younger caregivers

Identity-based disparities were another notable dimension of climate vulnerability. Bisexual or pansexual caregivers reported the highest levels of climate-related mental health burden, consistent with research documenting elevated psychological distress among bisexual adults that is linked to discrimination, invisibility, and erasure within both heterosexual and LGBTQ+ communities (Gonzales et al., 2016; Hafeez et al., 2017; Pollitt & Roberts, 2021). These patterns echo the broader lack of recognition that caregivers frequently face in policy and preparedness frameworks, indicating that caregivers identifying as bisexual or pansexual may contend with overlapping forms of marginalization.

Younger caregivers also reported elevated climate-related mental health burden, yet younger adults remain underexamined in caregiving policy and research despite their growing representation (Lacey et al., 2022). Because younger adults are more likely to identify as bisexual or pansexual (Pollitt & Roberts, 2021), these overlapping identities may help contextualize the patterns observed in this group. The climate-related mental health burdens reported may reflect a combination of identity-specific stressors and age-related disparities, highlighting the need to consider multiple axes of identity when assessing climate vulnerability.

### Implications for policy and future research

These findings point to several priorities for strengthening climate resilience in ways that better support caregivers. Future research should investigate how caregiving responsibilities intersect with structural disadvantages to shape climate-related mental health outcomes, using longitudinal and mixed-method designs to illuminate temporal dynamics and lived experiences. Studies that differentiate caregiving contexts, such as caregiving intensity, duration, or caregiver–recipient relationship, would also help identify which circumstances are most closely associated with reported climate-related mental health burden.

Caregiving extends beyond private responsibility to a role embedded in social and structural conditions, including policy gaps and broader systems of exclusion (Bom et al., 2019; Bouldin et al., 2018). Meaningfully integrating caregivers into climate resilience efforts requires moving beyond assumptions of individual preparedness to account for the contexts in which caregiving occurs. Many caregivers navigate climate disruptions while supporting individuals with complex care needs and do so with limited economic flexibility, unstable housing, or insufficient community infrastructure. Climate adaptation strategies that anticipate these realities—e.g., evacuation procedures and recovery resources designed with caregiving in mind—may help reduce the constraints caregivers face during extreme events. Strengthening the policy landscape, including more inclusive workplace protections and expanded financial relief, is similarly important for enabling caregivers to manage both routine responsibilities and climate-related disruptions.

These findings also highlight opportunities to improve support for bisexual, pansexual, and younger caregivers. Future work is needed to better understand how these groups mobilize support during climate events, including the role of chosen family and nontraditional kin networks that are central in many LGBTQ+ communities (Fredriksen-Goldsen et al., 2023). Policy efforts could expand paid caregiver leave to encompass broader definitions of family beyond biological or legally recognized ties, enabling caregivers who support friends, partners, or extended kin to access protections during climate disruptions (Heymann et al., 2024). Additional strategies might include developing caregiver-inclusive emergency communication tailored to younger adults and ensuring that climate resilience planning incorporates the caregiving structures present in diverse LGBTQ+ communities.

Attention to these structural determinants is essential for developing climate resilience strategies that support the well-being of caregivers and care recipients, particularly in communities already contending with social and economic disadvantages.

### Limitations

This study has several limitations. The cross-sectional design does not allow for causal inference, and climate exposure was measured through self-report, which may be subject to recall bias and lacks objective verification. Incorporating geospatial or environmental data in future research could strengthen exposure assessment. Future studies should also examine the impacts of specific climate events individually to better capture heterogeneity in caregivers’ challenges and psychological burdens, which may differ by event type and severity. Additionally, residual confounding from unmeasured variables, such as pre-existing psychological conditions or concurrent stressors, may have influenced the associations observed. We also did not account for variation in caregiving responsibilities, which may be associated with mental health burden in different ways.

Interpretation of these findings should also consider the ambiguity of the primary outcome measure. Because the survey item used adaptive wording based on household composition, reports of climate-related mental health burden may reflect the experiences of the caregiver, other household members, or both. This ambiguity introduces the possibility of nondifferential misclassification that could attenuate associations toward the null. Nonetheless, the consistency of associations across multiple structural and caregiving-specific factors supports the overall interpretability of the results.

## Conclusion

This study underscores the importance of centering caregiving within climate planning and public health preparedness. Caregivers, particularly those supporting older adults or belonging to marginalized communities, reported elevated climate-related mental health burden, highlighting the need for approaches that extend beyond individual readiness and acknowledge caregiving as a critical social role. Policymakers and practitioners can advance this goal by developing adaptation strategies that explicitly account for caregiver responsibilities, including improved access to mental health services and more robust local networks that help sustain caregiving during crises. Supporting caregivers is essential not only for their own well-being but also for maintaining the safety and continuity of care for those who depend on them. Building climate resilience with caregivers in mind is a necessary step toward more equitable, sustainable, and age-inclusive communities in a changing climate.

## Data Availability

This study was not pre-registered. Data used in this analysis are publicly available through the UCLA Center for Health Policy Research. Analytic methods and code are available from the corresponding author upon reasonable request.

## References

[gnag048-B1] Adger W. N. , Barnett J., Heath S., Jarillo S., Adger W. N., Barnett J., Heath S., Jarillo S. (2022). Climate change affects multiple dimensions of well-being through impacts, information and policy responses. Nature Human Behaviour, 6, 1465–1473. 10.1038/s41562-022-01467-8 36385175

[gnag048-B2] Amer Nordin A. , Mohd Hairi F., Choo W. Y., Hairi N. N. (2019). Care recipient multimorbidity and health impacts on informal caregivers: A systematic review. The Gerontologist, 59, e611–e628. 10.1093/geront/gny072 29982539

[gnag048-B3] Bailie J. , Matthews V., Bailie R., Villeneuve M., Longman J. (2022). Exposure to risk and experiences of river flooding for people with disability and carers in rural Australia: A cross-sectional survey. BMJ Open, 12, e056210. 10.1136/bmjopen-2021-056210 PMC9252212 35918120

[gnag048-B4] Bevans M. , Sternberg E. M. (2012). Caregiving burden, stress, and health effects among family caregivers of adult cancer patients. JAMA, 307, 398–403. 10.1001/jama.2012.29 22274687 PMC3304539

[gnag048-B5] Bom J. , Bakx P., Schut F., van Doorslaer E. (2019). The impact of informal caregiving for older adults on the health of various types of caregivers: A systematic review. The Gerontologist, 59, e629–e642. 10.1093/geront/gny137 30395200 PMC6850889

[gnag048-B6] Bouldin E. D. , Shaull L., Andresen E. M., Edwards V. J., McGuire L. C. (2018). Financial and health barriers and caregiving‐related difficulties among rural and urban caregivers. The Journal of Rural Health, 34, 263–274. 10.1111/jrh.12273 28940539 PMC5866208

[gnag048-B7] Broese van Groenou M. I. , De Boer A., Broese van Groenou M. I., De Boer A. (2016). Providing informal care in a changing society. European Journal of Ageing, 13, 271–279. 10.1007/s10433-016-0370-7 27610055 PMC4992501

[gnag048-B8] Butte County District Attorney’s Office. (2020). Camp fire public report: Summary of the camp fire investigation. https://www.buttecounty.net/DocumentCenter/View/1881/Camp-Fire-Public-Report—Summary-of-the-Camp-Fire-Investigation-PDF

[gnag048-B9] California Health Interview Survey. (2023). CHIS 2023 methodology series: Report 4 – Response rates. UCLA Center for Health Policy Research.

[gnag048-B10] California Health Interview Survey. (2024a). CHIS 2023 methodology series: Report 2 - Data collection methods. UCLA Center for Health Policy Research.

[gnag048-B11] California Health Interview Survey. (2024b). CHIS 2023 methodology series: Report 5 - Weighting and variance estimation. UCLA Center for Health Policy Research.

[gnag048-B12] Chase J. , Hansen P. (2021). Displacement after the camp fire: Where are the most vulnerable? Society & Natural Resources, 34, 1566–1583. 10.1080/08941920.2021.1977879

[gnag048-B13] Costello A. , Abbas M., Allen A., Ball S., Bell S., Bellamy R., Friel S., Groce N., Johnson A., Kett M., Lee M., Levy C., Maslin M., McCoy D., McGuire B., Montgomery H., Napier D., Pagel C., Patel J., Patterson C. (2009). Managing the health effects of climate change: Lancet and University College London Institute for Global Health Commission. Lancet (London, England), 373, 1693–1733. 10.1016/S0140-6736(09)60935-1 19447250

[gnag048-B14] Dennin L. R. , Nock D., Muller N. Z., Akindele M., Adams P. J., Dennin L. R., Nock D., Muller N. Z., Akindele M., Adams P. J. (2025). Socially vulnerable communities face disproportionate exposure and susceptibility to U.S. wildfire and prescribed burn smoke. Communications Earth & Environment, 6, 190. 10.1038/s43247-025-02100-y

[gnag048-B15] Fakeye M. B. K. , Samuel L. J., Drabo E. F., Bandeen-Roche K., Wolff J. L. (2023). Caregiving-related work productivity loss among employed family and other unpaid caregivers of older adults. Value in Health, 26, 712–720. 10.1016/j.jval.2022.06.014 35973924 PMC9922792

[gnag048-B16] Fazeli S. , Haghani M., Mojtahedi M., Rashidi T. H. (2024). The role of individual preparedness and behavioural training in natural hazards: A scoping review. International Journal of Disaster Risk Reduction, 105, 104379. 10.1016/j.ijdrr.2024.104379

[gnag048-B17] Fredriksen-Goldsen K. , Jen S., Emlet C. A., Kim H.-J., Jung H. H. (2023). Key determinants of physical and psychological health-related quality of life over time among midlife and older LGBTQ and sexual and gender-diverse caregivers. The Gerontologist, 63, 751–761. 10.1093/geront/gnac112 35933628 PMC10167761

[gnag048-B18] Gee G. C. , Payne-Sturges D. C. (2004). Environmental health disparities: A framework integrating psychosocial and environmental concepts. Environmental Health Perspectives, 112, 1645–1653. 10.1289/ehp.7074 15579407 PMC1253653

[gnag048-B19] Gonzales G. , Przedworski J., Henning-Smith C. (2016). Comparison of health and health risk factors between lesbian, gay, and bisexual adults and heterosexual adults in the United States: Results from the National Health Interview Survey. JAMA Internal Medicine, 176, 1344–1351. 10.1001/jamainternmed.2016.3432 27367843

[gnag048-B20] Hafeez H. , Zeshan M., Tahir M. A., Jahan N., Naveed S. (2017). Health care disparities among lesbian, gay, bisexual, and transgender youth: A literature review. Cureus, 9, e1184. 10.7759/cureus.1184 28638747 PMC5478215

[gnag048-B21] Heitmueller A. , Inglis K. (2007). The earnings of informal carers: Wage differentials and opportunity costs. Journal of Health Economics, 26, 821–841. 10.1016/j.jhealeco.2006.12.009 17276532

[gnag048-B22] Heymann J. , Raub A., Waisath W., Earle A., Stek P., Sprague A. (2024). Paid leave to meet the health needs of aging family members in 193 countries. Journal of Aging & Social Policy, 36, 508–531. 10.1080/08959420.2022.2110804 36007142

[gnag048-B23] Himes-Cornell A. , Ormond C., Hoelting K., Ban N. C., Koehn J. Z., Allison E. H., Larson E. C., Monson D. H., Huntington H. P., Okey T. A. (2018). Factors affecting disaster preparedness, response, and recovery using the community capitals framework. Coastal Management, 46, 335–358. 10.1080/08920753.2018.1498709

[gnag048-B24] Lacey R. E. , Xue B., McMunn A. (2022). The mental and physical health of young carers: A systematic review. The Lancet. Public Health, 7, e787–e796. 10.1016/S2468-2667(22)00161-X 36057277

[gnag048-B25] Lahaie C. , Earle A., Heymann J. (2013). An uneven burden: Social disparities in adult caregiving responsibilities, working conditions, and caregiver outcomes. Research on Aging, 35, 243–274. 10.1177/0164027512446028

[gnag048-B26] Lawrance E. L. , Thompson R., Vay J. N. L., Page L., Jennings N. (2022). The impact of climate change on mental health and emotional wellbeing: A narrative review of current evidence, and its implications. International Review of Psychiatry (Abingdon, England), 34, 443–498. 10.1080/09540261.2022.2128725 36165756

[gnag048-B27] Lindt N. , van Berkel J., Mulder B. C., Lindt N., van Berkel J., Mulder B. C. (2020). Determinants of overburdening among informal carers: A systematic review. BMC Geriatrics, 20, 304. 10.1186/s12877-020-01708-3 32847493 PMC7448315

[gnag048-B28] Liu Y. , Dokos M., Fauth E. B., Lee Y. G., Zarit S. H. (2019). Financial strain, employment, and role captivity and overload over time among dementia family caregivers. The Gerontologist, 59, e512–e520. 10.1093/geront/gnz099 31322654 PMC6857684

[gnag048-B29] Maranghides A. , Link E., Brown C. U., Walton W. D., Mell W., Hawks S. (2023). Supplement to: A case study of the Camp Fire—Notification, evacuation, traffic affic, and temporary refuge areas (NIST Technical Note 2252). National Institute of Standards and Technology. 10.6028/NIST.TN.2252

[gnag048-B30] Metzl J. M. , Hansen H. (2014). Structural competency: Theorizing a new medical engagement with stigma and inequality. Social Science & Medicine (1982), 103, 126–133. 10.1016/j.socscimed.2013.06.032 24507917 PMC4269606

[gnag048-B31] Meyer I. H. (2003). Prejudice, social stress, and mental health in lesbian, gay, and bisexual populations: Conceptual issues and research evidence. Psychological Bulletin, 129, 674–697. 10.1037/0033-2909.129.5.674 12956539 PMC2072932

[gnag048-B32] Nielsen K. S. , Cologna V., Bauer J. M., Berger S., Brick C., Dietz T., Hahnel U. J. J., Henn L., Lange F., Stern P. C., Wolske K. S., Nielsen K. S., Cologna V., Bauer J. M., Berger S., Brick C., Dietz T., Hahnel U. J. J., Henn L., … Wolske K. S. (2024). Realizing the full potential of behavioural science for climate change mitigation. Nature Climate Change, 14, 322–330. 10.1038/s41558-024-01951-1

[gnag048-B33] Peterson L. J. , Hackett S. E., Dobbs D., Haley W. E. (2024). Dementia caregivers’ perspectives on disaster preparedness: Barriers, resources, and recommendations. The Gerontologist, 64, gnad076. 10.1093/geront/gnad076 37351950

[gnag048-B34] Pickering C. J. , Dancey M., Paik K., O’Sullivan T., Pickering C. J., Dancey M., Paik K., O’Sullivan T. (2021). Informal caregiving and disaster risk reduction: A scoping review. International Journal of Disaster Risk Science, 12, 169–187. 10.1007/s13753-021-00328-8

[gnag048-B35] Pollitt A. M. , Roberts T. S. (2021). Internalized binegativity, LGBQ+ community involvement, and definitions of bisexuality. Journal of Bisexuality, 21, 357–379. 10.1080/15299716.2021.1984363 35185393 PMC8856634

[gnag048-B36] Riffin C. , Van Ness P. H., Wolff J. L., Fried T. (2017). Family and other unpaid caregivers and older adults with and without dementia and disability. Journal of the American Geriatrics Society, 65, 1821–1828. 10.1111/jgs.14910 28426910 PMC5555780

[gnag048-B37] Romanello M. , McGushin A., Di Napoli C., Drummond P., Hughes N., Jamart L., Kennard H., Lampard P., Solano Rodriguez B., Arnell N., Ayeb-Karlsson S., Belesova K., Cai W., Campbell-Lendrum D., Capstick S., Chambers J., Chu L., Ciampi L., Dalin C., Hamilton I. (2021). The 2021 report of the Lancet Countdown on health and climate change: Code red for a healthy future. Lancet (London, England), 398, 1619–1662. 10.1016/S0140-6736(21)01787-6 34687662 PMC7616807

[gnag048-B38] Sheehan O. C. , Haley W. E., Howard V. J., Huang J., Rhodes J. D., Roth D. L. (2021). Stress, burden, and well-being in dementia and nondementia caregivers: Insights from the Caregiving Transitions Study. The Gerontologist, 61, 670–679. 10.1093/geront/gnaa108 32816014 PMC8276607

[gnag048-B39] White B. P. , Breakey S., Brown M. J., Smith J. R., Tarbet A., Nicholas P. K., Ros A. M. V. (2023). Mental health impacts of climate change among vulnerable populations globally: An integrative review. Annals of Global Health, 89, 66. 10.5334/aogh.4105 37810609 PMC10558031

